# Supplementary material for: Aromatic oil from lavender as an atopic dermatitis suppressant
Source: PLoS One. 2024 Jan 5;19(1):e0296408. doi: 10.1371/journal.pone.0296408 (PMC10769034; doi:10.1371/journal.pone.0296408)
Supplement: S1 Raw images — The filenames are as follows: a. S1_raw_images_Figure3C.pdf b. 15.JPG c. 21.JPG d. 22.JPG e. 2201241741391.tif f. 2202041813141.tif g. 2202041817223.tif h. 2202041818381.tif i. 2202071847014.tif j. 2202071852122.tif k. 2202071853293.tif l. 2202071859374.tif m. S1_raw_images_FigureS2.pdf n. 7.JPG o. 8.JPG p. 2112201615449.tif q. 2112211700287.tif r. 2201132050057.tif s. Williams_test.txt t. grouped_scatter_plot_Bartlett_ANOVA_en.ipynb. (ZIP) [file S1_raw_images_FigureS2.pdf › S1_raw_14/S1_raw_images_FigureS2.pdf]

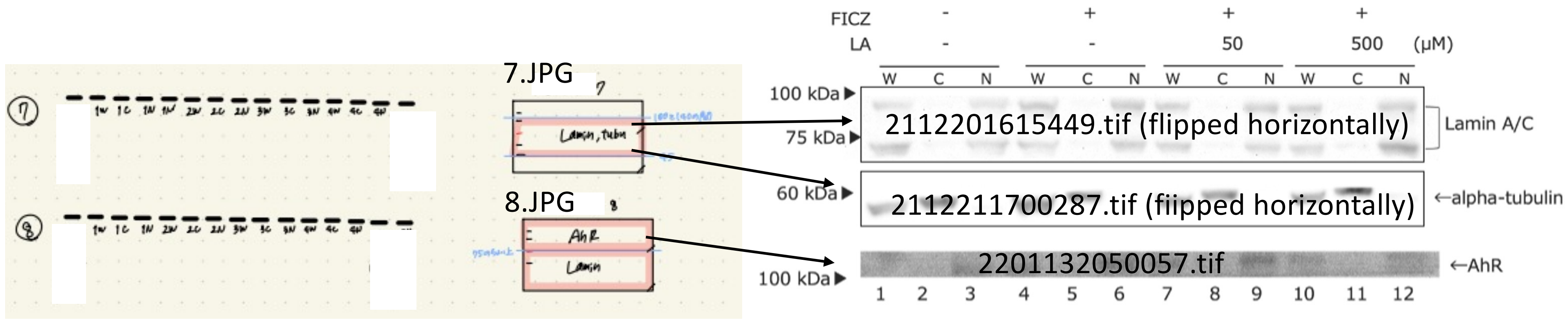

Supplementary Figure S2
