# Supplementary material for: Aromatic oil from lavender as an atopic dermatitis suppressant
Source: PLoS One. 2024 Jan 5;19(1):e0296408. doi: 10.1371/journal.pone.0296408 (PMC10769034; doi:10.1371/journal.pone.0296408)
Supplement: Attachment — Submitted filename: Response to Reviewers_PONE-D-23-13536.pdf [file pone.0296408.s004.pdf]

Reviewers' comments, Our answers

Reviewer #1:

Authors must go through very recent papers on how essential oils can find their applications in treating disorders, etc. Here are few references to cite. DOI: 10.3390/antiox11122410; DOI: 10.1016/j.jics.2021.100088, DOI: 10.1016/j.tifs.2022.10.012.

We are grateful for the enlightening insights shared in the recent papers. In the revised version of our manuscript, we have expanded our Introduction section in the context of the new applications of EOs by citing these papers.

Rest is fine and I recommend manuscript acceptance following the minor suggestions.

We are very grateful for the reviewer's positive evaluation of our manuscript and recommendation for acceptance.

Reviewer #2:

1. In the Introduction (Lines 46-47) the authors state that "It affects 15%–20% of children and 1%–3% of adults worldwide and patient numbers are increasing"- please provide the reference number for this epidemiological information.

We apologize for the inadvertent omission of the corresponding reference in our original submission and appreciate your diligence in identifying this oversight. We have now properly included the appropriate citation [1] in our revised manuscript (https://doi.org/10.1159/000370220).

2. In the same Introduction (Lines 65-66) the authors state that "Currently, steroids and immunosuppressive agents are used to treat AD. However, they are only symptomatic treatments". The authors should know that new drugs for AD have been recently authorized by FDA and EMA: dupilumab, baricitinb and tralokinumab (anti IL-13 agent) which can modulate specific pathogenetic mechanisms of AD. It is more appropriate to say that despite the recent developments in the pharmacotherapy of AD, there is still a real need for the discovery of new molecules capable of better controlling AD.

We appreciate your critical feedback. In response to your insightful suggestions, we have included the appropriate descriptions in the Introduction section of our revised manuscript.

3. In the Results section the authors state that "Both L. angustifolia essential oil and generic lavender oil inhibited AD". Please bear in mind that your study evaluated specific molecular events and not the extent of the real disease which can be evaluated in vivo (extent and progression of the lesions, etc). In fact the tested essential oils inhibited AhR activation not the disease itself which is a very complex entity.

We have revised the Results section to more accurately reflect that our study observed inhibition of AhR activation by both *L. angustifolia* essential oil and generic lavender oil, rather than direct inhibition of AD.
