# Supplementary material for: Aromatic oil from lavender as an atopic dermatitis suppressant
Source: PLoS One. 2024 Jan 5;19(1):e0296408. doi: 10.1371/journal.pone.0296408 (PMC10769034; doi:10.1371/journal.pone.0296408)
Supplement: S1 File — (PDF) [file pone.0296408.s002.pdf]

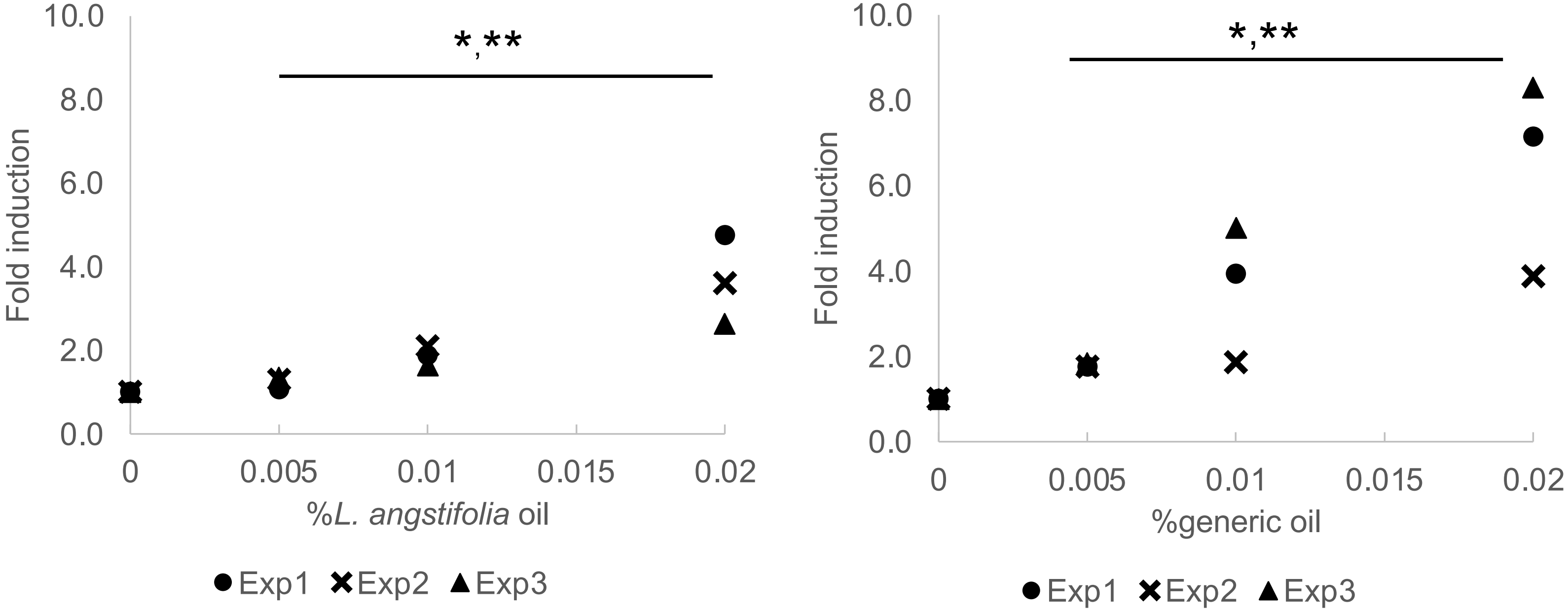

Supplementary Figure S1a

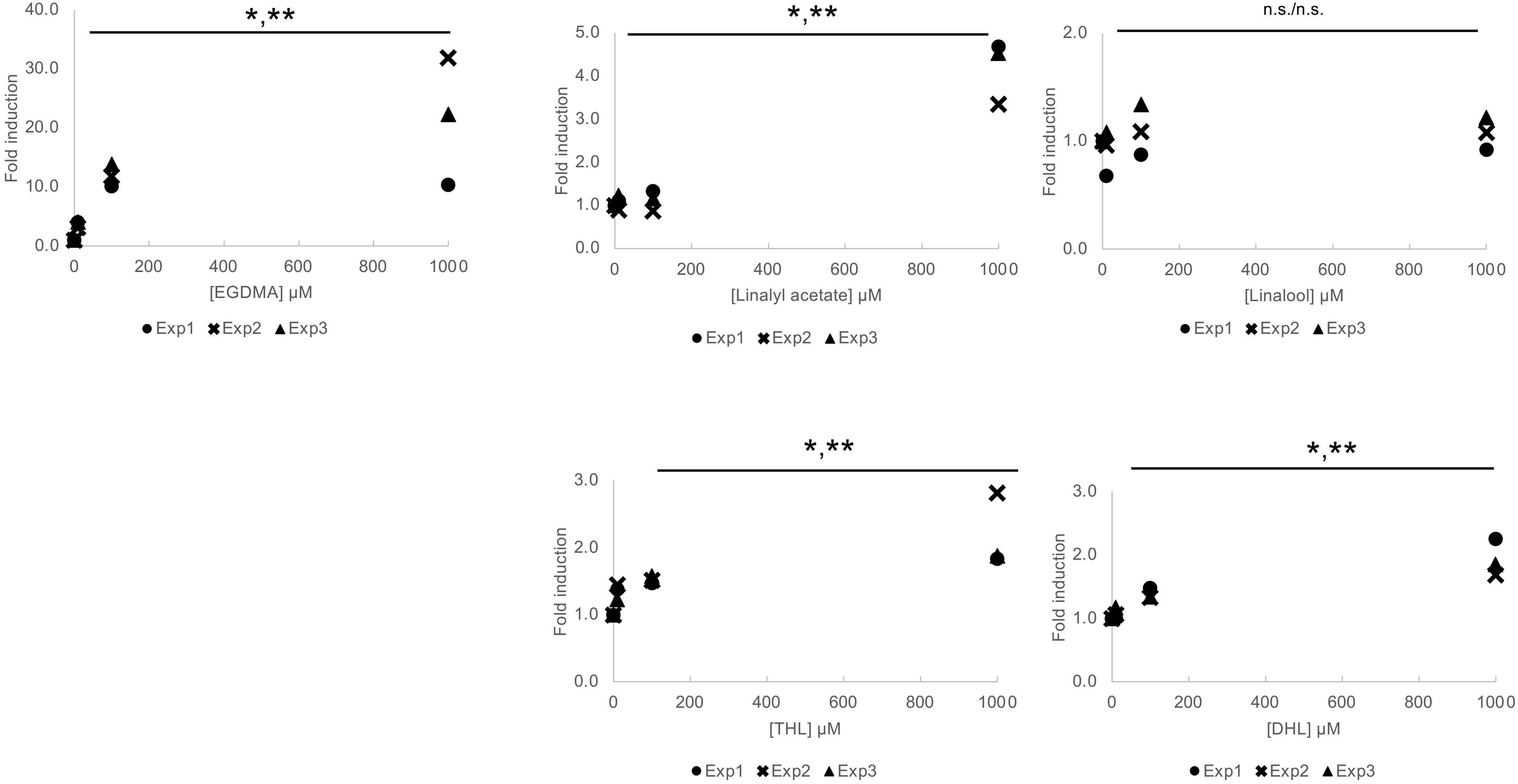

Supplementary Figure S1b

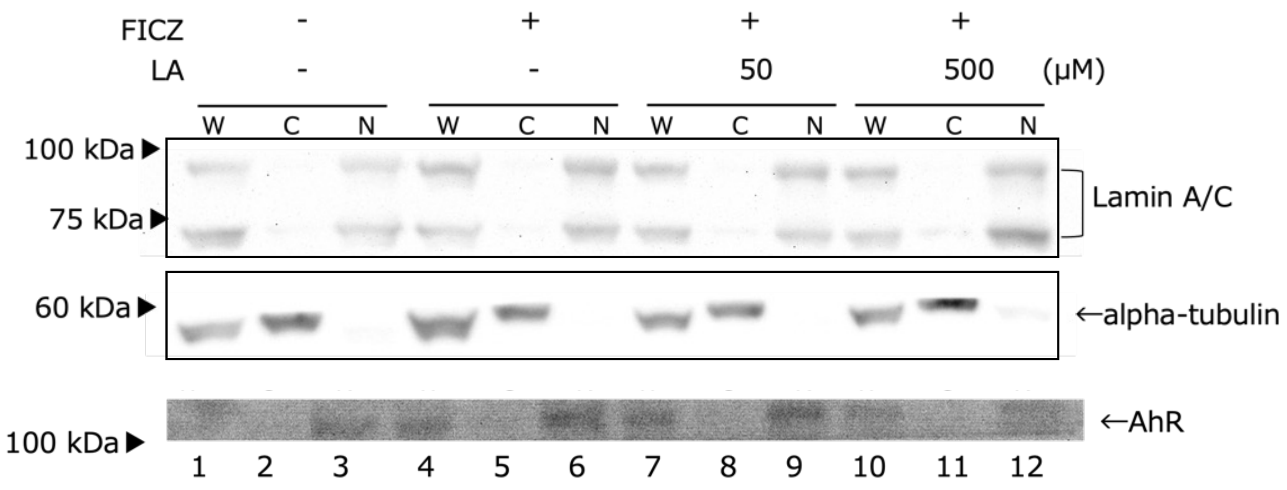

Supplementary Figure S2

Supplementary Table S1. Agents used in this study

name	Concentration (stock)	solvent	CAS#	Brand
Lavandula angustifolia essential oil	-	DMSO	-	Pranarom
Generic avender oil	-	DMSO	-	no brand
Linalool	5.56 M*	DMSO	78-70-6	Tokyo Chemical Industry Co., Ltd.
Linalyl acetate	4.56 M*	DMSO	115-95-7	Tokyo Chemical Industry Co., Ltd.
Tetrahydrolinalool	5.22 M*	DMSO	78-69-3	Tokyo Chemical Industry Co., Ltd.
6,7-Dihydrolinalool	5.50 M*	DMSO	18479-49-7	Santa Cruz Biotechnology
FICZ (6-Formylindolo[3,2-b]carbazole)	10 mM	DMSO	172922-91-7	Cayman Chemical
Ethylene glycol dimethacrylate	1 M	EtOH	97-90-5	Tokyo Chemical Industry Co., Ltd.
TNBS	-	RPMI+10%FBS	2508-19-2	Sigma-Aldrich
DL-lactic acid	-	RPMI+10%FBS	50-21-5	Sigma-Aldrich

*calculated by the following online calculator, https://www.omnicalculator.com/chemistry/percentage-concentration-to-molarity

Supplementary Table S2. Primer sets used in this study

gene	Forward primer	Reverse primer
GAPDH	5'-agccacatcgctcagacac-3'	5'-gcccaatacgaccaaatcc-3'
Artemin (ARTN)	5'-cgctcctggtgttgatagaga-3'	5'-gcggagagaccaagtgga-3'
AhR	5'-caacatcacctacgccagtc-3'	5'-gcttggaaggatttgacttga-3'
ARNT	5'-ctgtcatcctgaagaccagcag-3'	5'-ctggttctcatccagagccattc-3'

Supplementary Table S3. Composition analysis table of *L. angustifolia* oil used in this study

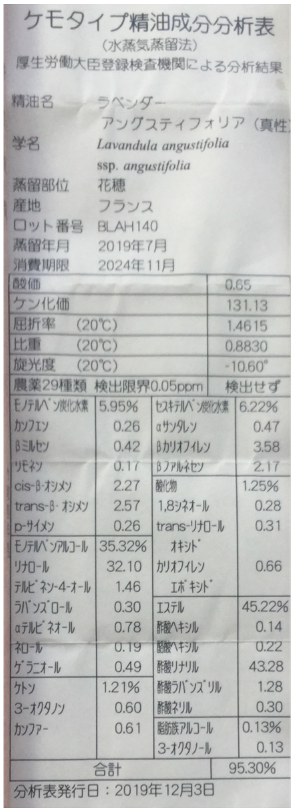

ケモタイプ精油成分分析表
（水蒸気蒸留法）
厚生労働大臣登録検査機関による分析結果

精油名　ラベンダー
　　　　アングスティフォリア（真性）
学名　*Lavandula angustifolia*
　　　ssp. *angustifolia*
蒸留部位　花穂
産地　フランス
ロット番号　BLAH140
蒸留年月　2019年7月
消費期限　2024年11月

酸価	0.65
ケン化価	131.13
屈折率　(20℃)	1.4615
比重　(20℃)	0.8830
旋光度　(20℃)	-10.60°
農薬29種類 検出限界0.05ppm	検出せず

モノテルペン炭化水素	5.95%	セスキテルペン炭化水素	6.22%
カンフェン	0.26	αサンタレン	0.47
βミルセン	0.42	βカリオフィレン	3.58
リモネン	0.17	βファルネセン	2.17
cis-β-オシメン	2.27	酸化物	1.25%
trans-β-オシメン	2.57	1,8シネオール	0.28
p-サイメン	0.26	trans-リナロールオキシド	0.31
モノテルペンアルコール	35.32%	カリオフィレンエポキシド	0.66
リナロール	32.10		
テルピネン-4-オール	1.46		
ラバンズロール	0.30	エステル	45.22%
αテルピネオール	0.78	酢酸ヘキシル	0.14
ネロール	0.19	酪酸ヘキシル	0.22
ゲラニオール	0.49	酢酸リナリル	43.28
ケトン	1.21%	酢酸ラバンズリル	1.28
3-オクタノン	0.60	酢酸ネリル	0.30
カンファー	0.61	脂肪族アルコール	0.13%
		3-オクタノール	0.13
合計			95.30%

分析表発行日：2019年12月3日

Essential Oil Composition Analysis Table

(Steam distillation method)
Analysis by an inspection organization registered with the Minister of Health, Labor and Welfare, in Japan

Essential oil name: Lavender
Angustifolia
Scientific name: *Lavandula angustifolia*
Distillation part: flower ear
Place of origin: France
Lot number: BLAH140
Distillation date: July 2019
Consumption date: November 2024

Acid value		0.65	
Saponification value		131.13	
Refractive index		1.4615	
Specific Gravity		0.8830	
Optical rotation		-10.60	
29 pesticides		Not detected	
Monoterpene hydrocarbons	5.95%	Sesquiterpene hydrocarbons	6.22%
camphene	0.26	alpha santalene	0.47
beta myrcene	0.42	beta caryophyllene	3.58
limonene	0.17	beta farnesene	2.17
cis-beta-ocimene	2.27		
trans-beta-ocimene	2.57		
p-cymene	0.26		
Monoterpene alcohols	35.32%	Oxides	1.25%
linalool	32.10	1, 8-cineole	0.28
terpinen-4-ol	1.46	trans-linalool oxide	0.31
lavandulol	0.30	caryophyllene epoxide	0.66
alpha terpineol	0.78		
nerol	0.19		
geraniol	0.49		
Ketones	1.21%	Esters	45.22%
3-octanone	0.60	hexyl acetate	0.14
camphor	0.61	hexyl butyrate	0.22
		linalyl acetate	43.28
		lavandulyl acetate	1.28
		neryl acetate	0.30
		Aliphatic alcohols	0.13%
		3-octanol	0.13
Total			95.30%
Date of Analysis Table Issued			December 3, 2019

Table S4. Raw data of the dendritic cell activation assay to calculate EC_{150} and CV_{70}

		%Viability	CD86⁺ FL1 positivity (%)	Isotype⁺ FL1 positivity (%)	S.I. (%)
culture medium	Experiment 1	98	9.6	1.3	
	Experiment 2	96.2	12.2	1.4	
DMSO	Experiment 1	97.7	22	0.8	
	Experiment 2	96.4	22.9	1.1	
TNBS 50 μg/mL	Experiment 1	96.4	34.2	7.7	320.3
	Experiment 2	94.9	47.9	4.6	399.9
lactic acid 200 μg/mL	Experiment 1	97.4	11.5	1.9	115
	Experiment 2	94.4	11.7	1.4	95.1
L. angustifolia essential oil 0.01%	Experiment 1	97.6	23	1.1	103
	Experiment 2	96.2	27.9	1.5	121.6
L. angustifolia essential oil 0.02%	Experiment 1	94.3	30.2	0.7	138.8
	Experiment 2	61.4	39.6	0.9	177.2
L. angustifolia essential oil 0.04%	Experiment 1	0.35	12.3	50	-177.3
	Experiment 2	0.041	33.4	33.3	0.5

		%Viability	CD86⁺ FL1 positivity (%)	Isotype⁺ FL1 positivity (%)	S.I. (%)
culture medium	Experiment 1	98	9.6	1.3	
	Experiment 2	97	9	0.8	
DMSO	Experiment 1	97.7	22	0.8	
	Experiment 2	94.9	16.8	0.2	
TNBS 50 μg/mL	Experiment 1	96.4	34.2	7.7	320.3
	Experiment 2	95.2	29.2	1.2	342.2
lactic acid 200 μg/mL	Experiment 1	97.4	11.5	1.9	115
	Experiment 2	96.4	8.4	0.7	94.4
generic lavender oil 0.01%	Experiment 1	96.6	23.7	1.1	103.8
	Experiment 2	96.2	21	0.4	124.1
generic lavender oil 0.02%	Experiment 1	94.8	28.3	1.2	124.1
	Experiment 2	94	25.9	0.3	154.1
generic lavender oil 0.04%	Experiment 1	86.4	40.4	1.2	179.8
	Experiment 2	81.4	42.9	0.5	255.5

		%Viability	CD86⁺ FL1 positivity (%)	Isotype⁺ FL1 positivity (%)	S.I. (%)
culture medium	Experiment 1	91.7	10.8	1.1	
	Experiment 2	96.7	12.5	1.1	
DMSO	Experiment 1	91.4	27.3	0.6	
	Experiment 2	95.9	22.1	0.6	
TNBS 50 μg/mL	Experiment 1	80.8	81	10.2	731.2
	Experiment 2	95.5	35.7	5.4	266.8
lactic acid 200 μg/mL	Experiment 1	96.2	10.1	1.1	93.3
	Experiment 2	95.4	12.6	1.4	98.6
LA 1 μg/mL	Experiment 1	97.1	23.8	1	85.3
	Experiment 2	96.5	18.9	0.7	84.7
LA 5 μg/mL	Experiment 1	94.2	23.9	0.9	86.3
	Experiment 2	95.1	20.2	0.7	91.1
LA 10 μg/mL	Experiment 1	86.2	29.7	2.8	101.1
	Experiment 2	87.6	24.5	1.1	109
LA 20 μg/mL	Experiment 1	12.7	38.5	3.7	130.6
	Experiment 2	69.6	22.3	0.5	101.5
LA 40 μg/mL	Experiment 1	0.91	85.5	35.7	186.6
	Experiment 2	-	-	-	-
