# Supplementary material for: Aromatic oil from lavender as an atopic dermatitis suppressant
Source: PLoS One. 2024 Jan 5;19(1):e0296408. doi: 10.1371/journal.pone.0296408 (PMC10769034; doi:10.1371/journal.pone.0296408)
Supplement: Attachment — Submitted filename: Response_to_reviewers_rev2.docx [file pone.0296408.s003.docx]

*Reviewer #1: This authors well-responded to reviewer's questions.
I have no further question.
Great job again for this manuscript.*

Thank you for taking the time to review our manuscript and for your kind words. We appreciate your support and comments.

*Reviewer #2: COMMENTS TO AUTHORS*
*This study describes the potential of L. angustifolia essential oil as an inhibitor of atopic dermatitis symptoms.* *However, the statistical analysis is insufficient, and it is unclear whether there are significant differences in Figures 1a, 2a, 2b, 3a, and 3b.*

Thank you for your comment regarding the statistical analysis in our manuscript. We apologize for any confusion that may have arisen. To address your concerns, we have carefully reanalyzed our data and performed appropriate statistical tests, specifically, the Williams test which is a nonparametric statistical procedure specifically designed to test for a significant dose-response trend in data. We appreciate your attention to detail and insight regarding Figures 3a and 3b. You are correct that these figures did not show significant differences, and we have accordingly labeled them "n.s." to indicate statistical insignificance. With respect to Figure 1a, we observed a significant difference, which was confirmed by both the Williams test and the Kruskal-Wallis test, an alternative to ANOVA for nonparametric data. We have taken your suggestion and updated the figure to include "*", "**" to indicate statistical significance. Regarding Figures 2a and 2b, we unfortunately lost the original raw data and had to re-run all the experiments. The results now show statistical significance as similar to those in Figures 1a and 1b. We believe that these changes and the improved statistical analysis provide a more transparent interpretation of our results. We sincerely thank you for your valuable feedback, which has undoubtedly strengthened our work.

*The results section should only include facts based on the data.*

We agree with you that it is important to present only facts based on the data. We have carefully reviewed our Results section and confirmed that it essentially contains only things necessary to understand the purpose of the experiments performed and the explanation of our data presented in figures and tables.

*Moreover, there are some concerns as mentioned below.
1. The description of time units is mixed between "h" and "hours," so please unify either of them.
2. Page 4, line 149: 10^5 => 105
3. Page 4, line 149: 10^6 => 106
4. Page 5, line 182:* *THUNDERBIRD® => THUNDERBIRD®
5. Page 5, line 192: MgCl2 => MgCl2
6. Page 5, line 206: 10^6 => 106*

We have corrected these errors in the revised manuscript.

*Reviewer #3: In this manuscript, Sato et al used cellular models to examine the effects of lavender essential oil and its major components including linalyl acetate, in inhibiting AhR activation and Artemin expression in human keratinocyte HaCaT cells, as well as their potential for skin sensitization (using HaCaT cells and human monocytes).*

*The authors must clarify for each dataset: n=? per group. The data should be presented as each point instead of a bar. The authors should also indicate how many independent experiments were reproduced. With such information, it is hard to justify the data themselves and the statistic.*

We have carefully reviewed and made sure that all figure legends indicate how many independent experiments were performed. We have modified the graphs as you suggested.

Original:

%inhibition

| %concentration | L. angstifolia oil | Generic lavender oil |
| --- | --- | --- |
| 0.005% | 12.129 | 2.64068 |
| 0.01% | 40.6639 | 23.8095 |
| 0.02% | 72.4908 | 43.1658 |

Revised:

%inhibition

| %generic lavender oil | Exp1 | Exp2 | Exp3 | --- Average |
| --- | --- | --- | --- | --- |
| 0 | 0 | 0 | 0 | 0 |
| 0.005 | 24.9154 | -12.6621 | -4.33117 | 2.64068 |
| 0.01 | 34.3895 | 23.5046 | 13.5343 | 23.8095 |
| 0.02 | 52.0692 | 36.5188 | 40.9093 | 43.1658 |

%inhibition

| % L. angustifolia oil | Exp1 | Exp2 | Exp3 | --- Average |
| --- | --- | --- | --- | --- |
| 0 | 0 | 0 | 0 | 0 |
| 0.005 | 16.1658 | 6.50035 | 13.7208 | 12.129 |
| 0.01 | 45.8786 | 28.0911 | 48.0218 | 40.6639 |
| 0.02 | 68.3329 | 68.7551 | 80.3843 | 72.4908 |

*The data on nuclear translocation should be shown, even the results are negative (line 328). It will help the authors’ discussion on the possibility of protein degradation.*

Thank you for your comment. We agree that including nuclear translocation data, even if negative, could provide valuable insight into the possibility of protein degradation. Therefore, we have included the corresponding data and its description as Supplemental figure S2 in the revised manuscript.

*The authors should carefully discuss about reports showing that Lavender and Linalyl acetate cause contact allergy.*

Thank you for your comment. We appreciate your concern regarding potential contact allergy caused by linalyl acetate. To address this concern, we performed RNAseq on linalyl acetate-treated HaCaT cells and found no evidence of contact allergy under the conditions tested by DisGeNET-Metascape, which is capable of visualizing changes that correlate with disease, including contact dermatitis. However, we acknowledge that there have been some studies implicating linalyl acetate in contact dermatitis. To clarify that our conclusion is limited, we have added some words in the revised Discussion section.

*TSLP is a key factor in AD. It has been shown (**Moon, 2018 Molecules) that TSLP production by Mast cells is reduced by Linalyl acetate. The authors should examine the TSLP expression and provide data on the effect of Lavender essential oil and linalyl acetate.*

Thank you for your comment. We acknowledge that TSLP is a critical cytokine in atopic dermatitis and is typically well expressed in certain types of immune cells. However, we found that the HaCaT cell line used in our study barely expresses TSLP unless its expression is induced by FICZ (our unpublished RNAseq and qPCR data). Therefore, it would be challenging to perform the experiment you suggest. We appreciate your suggestion and will keep it in mind for future studies. We have included a discussion of this issue in the revised Discussion section, along with the reference [33] you suggested.
